# Oxidative Stress in Cutaneous Lichen Planus—A Narrative Review

**DOI:** 10.3390/jcm10122692

**Published:** 2021-06-18

**Authors:** Simona Roxana Georgescu, Cristina Iulia Mitran, Madalina Irina Mitran, Ilinca Nicolae, Clara Matei, Corina Daniela Ene, Gabriela Loredana Popa, Mircea Tampa

**Affiliations:** 1 Department of Dermatology, Carol Davila University of Medicine and Pharmacy, 020021 Bucharest, Romania; simonaroxanageorgescu@yahoo.com (S.R.G.); matei_clara@yahoo.com (C.M.); tampa_mircea@yahoo.com (M.T.); 2 Department of Dermatology, Victor Babes Clinical Hospital for Infectious Diseases, 030303 Bucharest, Romania; drnicolaei@yahoo.ro; 3 Department of Microbiology, Carol Davila University of Medicine and Pharmacy, 020021 Bucharest, Romania; dr.gabriela.popa@gmail.com; 4 Cantacuzino National Medico-Military Institute for Research and Development, 011233 Bucharest, Romania; 5 Department of Nephrology, Carol Davila University of Medicine and Pharmacy, 020021 Bucharest, Romania; koranik85@yahoo.com; 6 Department of Nephrology, Carol Davila Clinical Hospital of Nephrology, 010731 Bucharest, Romania

**Keywords:** lichen planus, oxidative stress, antioxidants

## Abstract

Lichen planus (LP) is a chronic, immune-mediated inflammatory skin condition that mainly affects the skin (cutaneous LP, CLP) and oral mucosa (oral LP, OLP). However, the mechanisms involved in the pathogenesis of the disease are not fully elucidated. Over time, several theories that could explain the appearance of LP lesions have been postulated. The key players in LP pathogenesis are the inflammatory infiltrate consisting of T cells and the proinflammatory cytokines. The cytokines stimulate the production of reactive oxygen species that induce cell apoptosis, a defining element encountered in LP. The lead inquiry triggered by this revolves around the role of oxidative stress in LP development. There are currently numerous studies showing the involvement of oxidative stress in OLP, but in terms of CLP, data are scarce. In this review, we analyze for the first time the currently existing studies on oxidative stress in CLP and summarize the results in order to assess the role of oxidative stress in skin lesions offering a fresher updated perspective.

## 1. Introduction

Oxidative stress represents the disturbance of the balance between prooxidants and antioxidants in favor of prooxidants. Free radicals are small diffusible molecules containing one or more unpaired electrons in their external orbitals. Free radicals are highly reactive compounds because unpaired electrons tend to form pairs of electrons from other atoms [1,2]. Oxidation is defined chemically as the loss of an electron, while reduction is the gain of an electron. Thus, in chemical reactions, a free radical can act as an oxidizing agent, receiving an electron from other species, or as a reducing agent, by donating an electron to other species. Regarding the definition of oxidative stress, in recent years, more emphasis has been placed on the fact that it involves the alteration of redox signaling, the alteration of the prooxidant-antioxidant balance passing on in the background [3]. Reactive oxygen species (ROSs) include oxygen radicals but also non-radicals, which act as oxidizing agents or are easily converted to radicals when two free radicals react and share their unpaired electrons, non-radical results [4,5].

**Figure 1:**
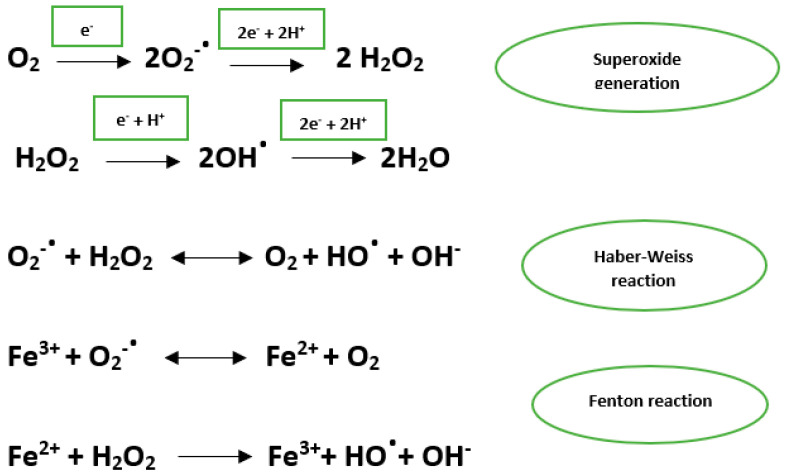
Superoxide generation through the reduction in oxygen and the generation of hydroxyl radical through Fenton and Haber Weiss reactions.

The main ROSs are the hydroxyl radical, superoxide anion, and hydrogen peroxide. The hydroxyl radical has a very short half-life and has the ability to act on most biological molecules. Oxygen superoxide is an important source of hydroxyl and hydrogen peroxide radicals [6] (Figure 1).

Oxidative stress may be involved in various disorders such as atherosclerosis, neoplasms, type II diabetes, neurodegenerative diseases, etc. [7,8]. Mitochondria are the primary source of oxidative stress. At the same time, the mitochondria have a defense system for detoxifying ROSs and repairing alterations caused by oxidative stress. Superoxide dismutases located in the mitochondrial matrix represent the main mitochondrial defenses against ROSs [9]. The redox centers in the mitochondria are organized into four protein complexes (complexes I, II, III, and IV) located in the inner mitochondrial membrane. The main ROS generation sites are located in complexes I and III. At the cellular level, there are many other sources of ROS production, including the endoplasmic reticulum, peroxisomes, lysosomes, and cytosol [10]. Peroxisomes, due to their catalase content acting on hydrogen peroxide, play an important role in maintaining the balance between oxidants and antioxidants [11]. However, peroxisomes may participate in the production of ROSs via β-oxidation of the fatty acids [12,13].

ROSs play a dual role in the human body as destructive and constructive species. Under physiological conditions, ROSs act as second messengers in various signaling pathways and are involved in the defense against infectious agents. ROSs activate sensitive redox transcription factors such as nuclear factor-kB (NF-kB), modulate cell differentiation, senescence and survival as well as regulate the expression of proinflammatory cytokines, etc. [12]. Interestingly, there are various processes that protect cells from the action of oxidative stress, mediated even by ROSs, with the role of restoring redox homeostasis at the cellular level. They are involved in the induction of cell apoptosis, thus having an anticarcinogenic role, but on the other hand, we must not forget the important involvement, supported by numerous studies of oxidative stress in the induction of neoplasms [14]. When ROSs accumulate and the antioxidant mechanisms are overwhelmed, oxidative stress exerts harmful effects on cell structures, such as lipids, proteins, carbohydrates, and nucleic acids [5,15].

Lipid peroxidation is the process that involves the oxidation of lipids, especially polyunsaturated fatty acids, which have a double carbon bond in their structure by free radicals or non-radical species. Under conditions of increased lipid peroxidation, the cells lost their ability to repair the damage caused, and apoptosis is induced [15]. The primary products of lipid peroxidation are unstable compounds, hydroperoxides that will lead by decomposition to stable molecules such as aldehydes, the most studied aldehydes being the malondialdehyde (MDA), 4-hydroxynonenal (4-HNE), and isoprostane [16].

Under conditions of oxidative stress, ROSs oxidize proteins resulting in structural and functional changes (e.g., the proteins may lose amino acids from their structure or may be fragmented) and increased levels of carbonyl groups. There are some amino acids that are more susceptible than others, including tryptophan, tyrosine, and cysteine. For example, the interaction between cysteine and ROSs leads to the generation of disulfides and oxyacids [17].

DNA oxidation induces changes in purine and pyrimidine bases and the formation of cross-linked bonds with other molecules. Mitochondrial DNA, which plays an essential role in the functioning of the mitochondria and the whole cell, is one of the main targets of ROSs [18,19]. Repair mechanisms are activated; however, in some instances, the damage cannot be prevented. Thus, these changes in the DNA structure have a mutagenic effect contributing to the development of malignant and aging processes [20]. Guanine is the most susceptible to oxidation among the nucleobases. 8-hydroxy-2′-deoxyguanosine (8-OHdG) formation is the most common and abundant change that occurs in the DNA structure [21].

Advanced glycation end products (AGEs) result from non-enzymatic glycation reactions. Reduced sugars react non-enzymatically with amino acids in the structure of proteins, lipids, and nucleic acids. These glycation compounds undergo rearrangements and condensations, resulting in irreversible products, which have been called AGEs, a group of highly oxidant, biologically active compounds. If they accumulate, they exert harmful effects on cellular structure and function. The main mechanism by which AGEs interfere with biological functions is the activation of specific transmembrane receptors for advanced glycation end-products [22,23].

The skin is the main target of ROSs [24]. Oxidative stress is an intrinsic part of skin metabolism. In the skin, under normal conditions, small amounts of ROSs are produced, which contribute to processes such as proliferation, differentiation, and cell apoptosis. The main exogenous factors that increase the production of ROSs in the skin are ultraviolet radiation, cigarette smoke, various pollutants, exposure to different microorganisms, etc. Inflammatory processes and psychological stress are part of the endogenous factors that can lead to an altered balance between prooxidants and antioxidants [24,25]. In this context, antioxidant systems play a defining role in counteracting the effects of oxidative stress [25]. In the skin, both enzymatic and non-enzymatic antioxidants are involved in the defense against ROSs. The level and activity of antioxidant agents are higher in the epidermis compared to the dermis [26]. In the skin, the accumulation of ROSs leads to the development of a chronic inflammatory process, fragmentation, and disorganization of collagen fibers, resulting in significant alterations in the functional status of the cell, changes that can underlie in some cases the development of a malignant process. High levels of ROSs in the skin cause erythema, edema, and pain. In the development of skin diseases, oxidative stress may play the role of initiator of the pathogenic processes responsible for the appearance of the disease or may be the result of the activity of inflammatory cells involved in pathogenesis [5,25].

Lichen planus (LP) is a chronic mucocutaneous disease, immune-mediated, with unknown etiology, with global distribution, and no major familial predisposition has been identified. In terms of sex distribution, women are more frequently affected than men1. Most cases start between the 3rd and the 6th decades of life. LP affects around 0.5–1% of the general population [27,28].

**Figure 2:**
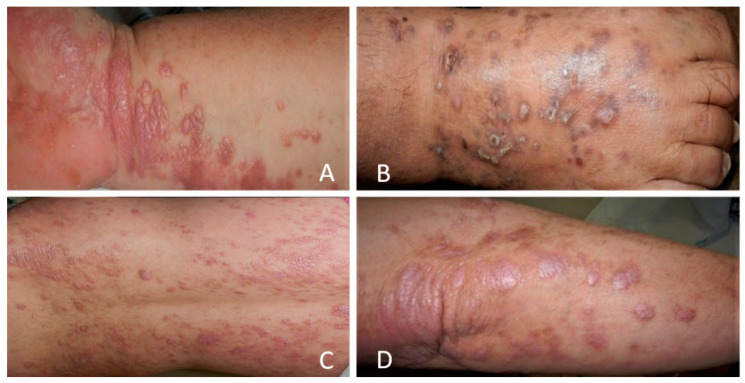
Clinical aspect of CLP. Small, polygonal, flat-topped, violaceous papules that may coalesce into plaques (**A**)—plaques on the wrist, (**B**)—dorsal aspect of the foot, (**C**)—disseminated papules and plaques on the back, (**D**)—plaques on the elbow.

LP more frequently involves the skin (cutaneous LP) and oral mucosa (oral LP). Other regions that may be involved are the scalp, nails, and the mucous membranes of the genitalia. Cutaneous LP (CLP) can be the sole clinical presentation of the disease. The elementary lesion in CLP is a pruritic well-demarcated violaceous polygonal papule, with white streaks on its surface, forming a network (Wickham network) (Figure 2). The papules can coalesce into plaques or placards, being located specially on the flexor surfaces of the extremities [29,30].

The histopathological features of CLP include epidermal changes, orthokeratosis accompanied by irregular acanthosis (“sawtooth” appearance), and hypergranulosis. In addition, liquefaction degeneration can be observed in the cells of the basal layer. In the dermis, a dense band-like chronic inflammatory cell infiltrate composed mainly of lymphocytes is present. Civatte bodies (necrotic keratinocytes) are characteristic of CLP and are located in the papillary dermis but can also appear in the lower part of the epidermis [31,32].

The pathogenesis of CLP is not fully elucidated. The central phenomenon underlying the appearance of CLP lesions is the cell-mediated immune response in basal keratinocytes, which subsequently become a source of antigens that can trigger the immune response [33]. The main cells involved are helper T cells, cytotoxic T cells, natural killer cells (NK), and dendritic cells. The most important role is attributed to CD8+ T cells, which infiltrate the epithelium, leading to the apoptosis of the basal layer keratinocytes [30]. It is assumed that the keratinocytes in the lesions express an LP antigen (not yet identified). The antigen is considered to be either a peptide of its own or a heat shock protein. Dysregulation of the gene responsible for the expression of heat shock proteins in the epithelial cells may interfere with the recognition of these proteins by the immune response resulting in an autoimmune process [34]. It is thought that in a proinflammatory environment, keratinocytes express heat shock proteins. The main mechanisms that could contribute to keratinocyte apoptosis, a histopathological hallmark and a telltale sign in CLP, are the secretion of tumor necrosis factor (TNF) alpha that binds to the keratinocyte receptor, the release of granzymes following the activation of cytotoxic T lymphocytes or the activation of pro-apoptotic signaling pathways [35].

More and more studies attribute an important role to oxidative stress in the pathogenesis of skin diseases with an autoimmune component such as alopecia areata, pemphigus vulgaris, psoriasis, vitiligo, etc. [36,37,38,39,40]. Regarding LP pathogenesis, there are numerous studies that emphasize the role of oxidative stress, but most of them include patients with OLP. The question that arises is whether oxidative stress plays an important role in the appearance of skin lesions as in OLP. This review aims to present and discuss the role of oxidative stress in CLP, offering a fresher and updated perspective on this topic.

## 2. Materials and Methods

We have performed a narrative review by browsing the PubMed and Google Scholar databases. The search strategy used keywords such as “oxidative stress lichen planus” and “antioxidants lichen planus”. The inclusion criteria were articles written in English, original articles, studies that included patients with CLP. We excluded reviews, clinical cases, and studies that evaluated patients with other forms of LP (oral, pilar, genital, and nail variants). After applying these criteria, we have identified 13 original articles. Due to the great heterogeneity of methods, materials, and results of the analyzed studies, a meta-analysis was not deemed appropriate.

## 3. Results and Discussions

**Figure 3:**
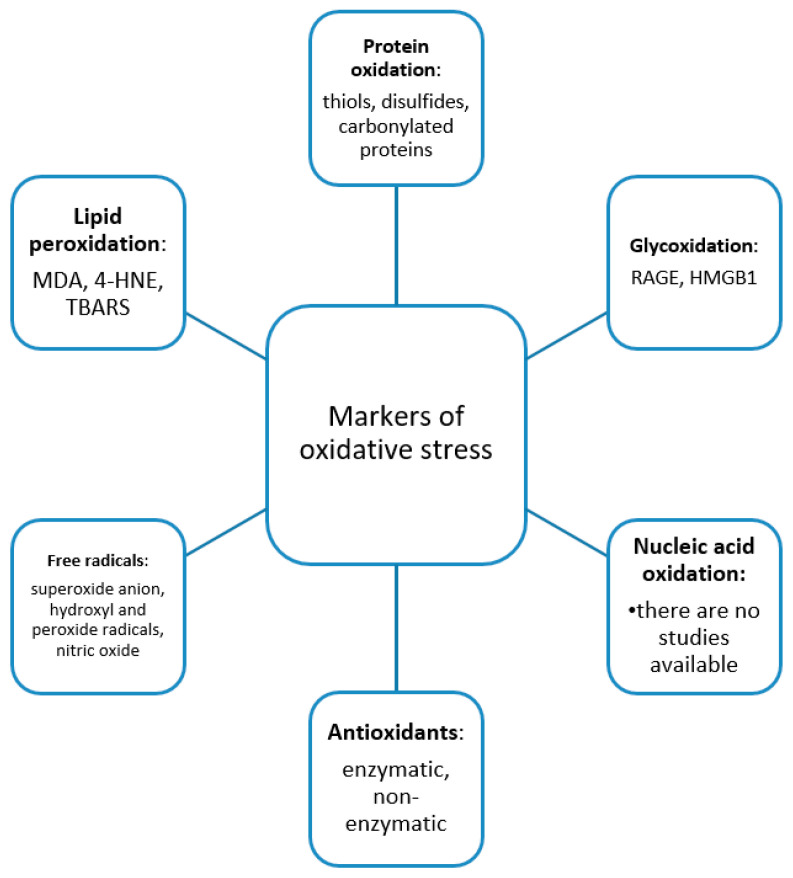
Summary of the oxidative stress markers evaluated in CLP patients, in the studies we have identified, encompassing the categories: markers of lipid peroxidation, oxidation of carbohydrates, proteins and nucleic acids, antioxidants, and free radicals.

A summary of the main data of the studies included in the review is presented in Table 1. The markers of oxidative stress evaluated in the studies, classified according to the oxidative stress targets, are presented in Figure 3.

We have found only studies between 2007 and 2019. Most of them being published in the last decade (2011–2019), which shows that there is a recent focus on this topic.

The studies are heterogeneous and investigate different markers of oxidative stress, which can be classified into five categories, markers of lipid peroxidation, oxidation of carbohydrates, and oxidation of proteins, antioxidants, and free radicals. We have not identified any study that evaluated nucleic acid oxidation. There is also heterogeneity in terms of the method used; most researchers have made the determinations in patient serum; however, there are studies in which the determinations were made in tissue samples. Most studies included a small number of patients. Therefore, a meta-analysis was not deemed appropriate.

Immunohistochemical examinations revealed that a dense inflammatory infiltrate composed of CD4+ T cells is present in CLP and represents a source of ROSs. ROSs activate the NF-kB signaling pathway resulting in increased expression of E-selectins, intercellular adhesion molecule (ICAM)-1 and vascular cell adhesion molecule (VCAM)-1, molecules that participate in the migration of lymphocytes to the site of inflammation and the formation of intradermal perivascular infiltrates, observed in LP [41]. In addition, the released proinflammatory cytokines will stimulate keratinocytes to produce ROSs [54]. The characteristic feature of LP, keratinocyte apoptosis, accompanied by liquefaction of the basal cell layer, may be the result of the cytokines released from the inflammatory infiltrate [34]. It has been shown that ROSs reduce the expression of p53, an important inhibitor of apoptosis. Another mechanism by which ROSs induce apoptosis is through the Fas-FasL pathway. It has been observed that in the presence of high levels of antioxidants such as glutathione, Fas-mediated apoptosis is inhibited [55]. TNF alpha-induced apoptosis is also mediated by ROSs. Overexpression of antioxidant enzymes inhibits the activation of TNF alpha-mediated signaling pathways and the activation of caspases, proteases essential in apoptosis [56]. Petti et al. hypothesized that oxidative stress could underlie the interrelationship between LP and hepatitis C virus (HCV) [57]. Breaking the balance between prooxidants and antioxidants can lead to an altered immune response against HCV infection [58].

Direct measurement of ROSs is difficult. Most studies are performed in vitro and focus on the assessment of products resulting from lipid peroxidation, protein oxidation, and DNA oxidation [59]. Determination of oxidative stress markers is most often performed using blood or urine samples. Depending on the molecular target of ROSs, the oxidative stress markers exhibit a great variety [60].

### 3.1. Lipid Peroxidation in CLP

Lipid peroxidation was investigated in six of the analyzed studies, including a total of 256 patients. In all studies, the authors made the determinations in patient serum and measured MDA as a marker of lipid peroxidation, and identified higher levels compared to the control group. Karim et al. determined serum and tissue MDA and identified lower levels in CLP patients compared to controls. They also revealed lower levels of serum and tissue CAT when compared to the groups, which highlights the oxidant-antioxidant imbalance at the cellular level. This imbalance was more pronounced in men, which can be explained by the antioxidant effect of estrogens in women [45]. Only one study measured the levels of 4-HNE and TBARS and revealed higher levels in CLP patients compared to the control group.

MDA and 4-HNE are among the most important reactive carbonyl species resulting from lipid peroxidation [53]. Lipid peroxidation products such as MDA and 4-HNE can alter cellular homeostasis. Membrane lipid peroxidation induces a cellular response, stimulates cell proliferation, interacts with membrane receptors and transcription factors, and promotes the activation of both the intrinsic and extrinsic signaling pathways governing apoptosis. The balance between pro-inflammatory and anti-inflammatory lipid peroxidation products is of particular importance in maintaining skin homeostasis and modulating the inflammatory process [61,62].

MDA generated during lipid peroxidation is a highly reactive compound, which induces changes in proteins through alterations in amino acids, resulting in the generation of neo-epitopes, which may trigger an immune response with the production of autoantibodies [63]. That is why MDA could play a role in the pathogenesis of autoimmune diseases. It should be noted that one of the most important theories proposed in the pathogenesis of LP is autoimmunity [27]. A recent study has revealed that LP patients have autoreactive peripheral blood Th17 cell responses against bullous pemphigoid (BP) 180, the autoantigen of BP [64]. Moreover, IL-17 promotes the release of pro-inflammatory cytokines leading to the recruitment of T cells, which are critical immune cells in the pathogenic mechanisms of LP [65]. The role of MDA in autoimmunity is also supported by a recent study that included patients with systemic lupus erythematosus and highlighted a positive correlation between anti-MDA IgG titer and markers of systemic inflammation. MDA is also involved in carcinogenesis, interfering with the cell cycle and apoptosis [66].

4-HNE interferes with cellular functions by producing adducts with proteins involved in signaling processes and regulates the expression of enzymes such as kinases, phosphatases, etc. [63]. In addition, 4-HNE adducts may have immunogenic properties as in the case of MDA. 4-HNE is involved in activating extrinsic pathways of cellular apoptosis, modulating the expression of the transcription factors NF-kB and AP-1 [67]. NF-kB mediates the expression of cytokines such as IL-2 and TNF alpha. In turn, TNF alpha stimulates the production of ROSs in epidermal keratinocytes. Oxidation processes of lipid-rich structures lead to the alteration of their function resulting in the promotion of a pro-inflammatory status and abnormal cell proliferation, processes also observed in LP [42].

### 3.2. Nucleic Acid Oxidation in CLP

Regarding nucleic acid oxidation, we did not identify studies that analyzed this process in CLP patients. Sander et al. have found higher levels of 8-OHdG in tissue harvested from patients with vulvar LP compared to healthy subjects [54]. We have not included this study in our review, given that we have included only studies evaluating patients with CLP. Nucleic acid oxidation was also investigated in patients with OLP. One of the most important complications of OLP is the transformation of the oral lesions into squamous cell carcinoma (SCC) [68]. It is well known that free radicals interact with cell components leading to tissue damage that can underlie the development of a malignant process. Elevated levels of 8-OHdG have been identified in malignant cells. Tumor cells are involved in the generation of ROSs that subsequently leads to the formation of 8-OHdG [69]. Nandakumar et al. investigated salivary 8-OHdG in patients with oral SCC, patients with oral submucous fibrosis, a premalignant condition, and healthy individuals. There were significant differences between patient groups and healthy controls, and the highest levels of 8-OHdG were detected in patients with oral SCC. They concluded that 8-OHdG could be regarded as a biomarker of DNA damage to evaluate disease progression [70]. 8-OHdG is the most widely used marker of oxidative stress resulting from DNA oxidation [71,72]. The formation of 8-OHdG on DNA is associated with mutations that promote carcinogenesis, cell aging, and certain degenerative disorders [21]. We consider that it would be interesting to explore nucleic acid oxidation in CLP.

### 3.3. Carbohydrate Oxidation in CLP

We have identified only one study that evaluated carbohydrate oxidation; de Carvalho et al. investigated the level of RAGE in skin tissue from CLP patients and healthy subjects. RAGE can be considered as an indirect marker of carbohydrate oxidation. They concluded that increased levels of RAGE in the dermis in CLP patients could play a role in disease pathogenesis [49]. RAGE is involved in numerous signaling pathways, modulating the transcription of cytokines, chemokines, matrix metalloproteinases, adhesion molecules and participates in cell proliferation, differentiation, and migration. For example, when S100B binds to RAGE activates protein kinase B and the NF-κB pathway and induces cell proliferation [73]. One of the main ligands of RAGE is high mobility group box protein 1 (HMGB1), a member of the family of AGEs; de Carvalho et al. have found high levels of HMGB1 in the dermis of LP lesions. The same results were observed for TLR-4. HMGB1 is one of the main DAMPs (damage-associated molecular patterns); therefore, these results may explain the role of HMBG1 in triggering the inflammatory process in LP [49]. The dynamics of the glycation process can be involved in many diseases, as evidenced by numerous recent researches that attribute to the AGEs-RAGE axis an important role in various pathologies [74]. The formation of AGEs by glycoxidation involves the participation of ROSs. The generation of the superoxide anion or hydroxyl radical results in the formation of glyoxal and methylglyoxal. These compounds will react with various biomolecules generating AGEs [74].

### 3.4. Protein Oxidation in CLP

Two studies evaluated protein oxidation in patients with CLP and used markers thiol disulfide homeostasis parameters (native thiol, total thiol, and disulfide) [52,53]. Kalkan et al. analyzed thiol disulfide homeostasis in 81 patients with CLP. NT and TT levels were statistically significantly higher in patients with CLP compared to the control group; however, no differences were identified in terms of disulfide levels. A positive association between the levels of glutathione, one of the most important low molecular weight thiols in cells, and cell proliferation has been found. Histopathological characteristics of LP such as orthokeratosis, accompanied by irregular acanthosis (“sawtooth” appearance) and hypergranulosis denote that CLP may be regarded as a proliferative disorder [52]. However, in a previous study, we found significantly lower levels of NT and TT and increased levels of DS in patients with CLP compared to the control group. These results may be the consequence of increased levels of pro-oxidants that exhausted the antioxidant systems [53].

The conversion of thiols to disulfides is a marker of protein oxidation [75]. In recent years, thiol-disulfide homeostasis has been studied in various dermatological conditions. Different results have been obtained, but all attest to the role of thiols and disulfides in the oxidant-antioxidant balance and subsequently in disease pathogenesis [76,77,78]. Thiols are an important part of the antioxidant system playing an essential role in mechanisms such as apoptosis, activation of signaling pathways, detoxification, etc. [79]. Thiols act as antioxidants due to their reducing properties. ROSs transfer excess electrons to thiols, resulting in thiol oxidation and the generation of disulfide bonds. Disulfides are degraded according to the oxidant-antioxidant balance, which makes this process dynamic [80]. The increased consumption of thiols and a high pro-oxidant potential of the serum lead to cell damage and could be a key step in the onset and progression of LP lesions. Under oxidative stress conditions, many processes, including cell proliferation, apoptosis, detoxification, and antioxidant defense, are altered [53].

### 3.5. Antioxidants in CLP

Antioxidant systems prevent the formation of ROSs, block their activity or act as scavengers for free radicals. On the other hand, antioxidant systems repair the alterations caused by the action of ROSs in various cellular components; therefore, DNA damage is corrected through enzymes, oxidized proteins are removed by the proteolytic systems, and phospholipases and acyltransferases repair the oxidized lipids [81]. Abdolsamadi et al. stated that the low level of antioxidants might be seen as a risk factor for the occurrence of OLP [82].

Most of the studies included in this review have focused on determining antioxidant markers in order to assess the imbalance between prooxidants and antioxidants. Enzymatic antioxidants (superoxide dismutase-SOD, catalase-CAT, glutathione peroxidase-GPX) were more frequently evaluated. SOD and CAT enzymes were determined in LP patients’ serum and in erythrocytes and as well as in LP lesions. In all studies that investigated SOD activity, increased activity was identified. In contrast, CAT activity was lower, and GPX activity was lower, or no significant differences were identified compared to the control group. These results show the imbalance of antioxidant systems. CAT is the main enzyme involved in the removal of H_2_O_2_; therefore, in the context of low levels of CAT and high levels of SOD that converts the superoxide anion into H_2_O_2_, there is an excessive accumulation of H_2_O_2,_ which will lead to the vacuolization of basal cells in LP lesions [41].

Regarding non-enzymatic antioxidants, in all studies, diminished or similar levels were identified in CLP patients compared to the control group. Another important component of the antioxidant systems is the uric acid that can neutralize ROSs and bind metal ions that will not be able any longer to catalyze various ROS-generating reactions [83]. Barikbin et al. investigated the serum levels of antioxidant compounds such as GPX, vitamin C, selenium, bilirubin, and uric acid in patients with CLP and healthy subjects and revealed significant differences between the two groups only for vitamin C. An explanation could be the fact that vitamin C is water-soluble and represents one of the primary antioxidants against oxidative stress. The authors pointed out that vitamin C supplements may be useful in the management of CLP [44].

### 3.6. Reactive Oxygen and Nitrogen Species in CLP

The study by Sapuntsova et al. is the only one that evaluated the level of oxygen radicals in LP lesions. Sapuntsova et al. analyzed biopsies from patients with LP and atopic dermatitis in terms of the proliferative process and free radical formation. They observed increased proliferation in LP and atopic dermatitis compared to normal skin, slightly lower in atopic dermatitis compared to LP, and high levels of oxidative stress in both diseases. The patients were treated for 10 days with thymodepressin, an immunosuppressant, and a decrease in the proliferative process and the level of oxidative stress were observed. This study demonstrates that alterations in cell differentiation may occur in the context of increased cell proliferation and oxidative stress [43].

Regarding reactive nitrogen species, the central role is played by the free radical NO^−^, which is formed by the conversion of L-arginine to citrulline, a reaction catalyzed by nitric oxide synthase (NOS) [84]. In all four studies evaluating NO, including 175 patients, higher levels were identified in CLP patients compared to the control group. NO can increase the proliferation of CD4 T cells that are identified in LP lesions and inhibit their apoptosis leading to cell accumulation. Thus, it can be seen as a compound that mediates the proliferation and differentiation of T cells [85,86].

## 4. Clinical Applications

The results of the analyzed studies provide evidence that there is an alteration of the balance between oxidants and antioxidants in patients with CLP. Elevated levels of ROSs lead to an inflammatory process and tissue damage that can contribute to the development/progression of CLP lesions. There are few studies that have looked at whether there is a correlation between CLP duration and oxidative stress status. Aly et al. found a positive correlation between NO, MDA, and SOD levels and the duration of CLP and a negative correlation between CAT levels and disease duration. Chakraborti et al. identified a negative correlation between uric acid levels and disease duration. Thus, the lowest uric acid levels were detected in patients with a duration of the disease that exceeded 6 months. Chakraborti et al. suggested that uric acid can be used as a biomarker of oxidative stress in the therapeutic approach and monitoring of CLP patients [47].

There are several studies that have evaluated antioxidant agents in the treatment of patients with OLP [87]. Abdeldayem et al. performed a randomized controlled clinical trial on patients with OLP. They divided the patients into two groups (patients receiving topical triamcinolone acetonide adhesive paste and vitamin E and patients receiving topical triamcinolone acetonide adhesive paste and placebo). The patients receiving vitamin E experienced greater pain reduction and clinical improvement than those receiving placebo. These results show that vitamin E may be useful in the treatment of OLP [88]. Suvarna et al. conducted a study in order to compare the efficacy of oral zinc 50 mg and 0.1% triamcinolone Orabase with 0.1% triamcinolone Orabase alone on the healing of OLP lesions and concluded that zinc may be a useful adjuvant in the therapy of OLP. Zinc administration relieved the burning sensation and reduced the lesion size [89].

## 5. Conclusions

There is an imbalance between oxidants and antioxidants in patients with CLP. Regarding the molecular targets of oxidative stress, most studies have evaluated lipid peroxidation, identifying elevated levels. Studies evaluating protein oxidation have highlighted the alteration of thiol-disulfide homeostasis in LP. Data on carbohydrate oxidation are scarce, and DNA oxidation was not evaluated in any study. Most studies have revealed low levels of both enzymatic and non-enzymatic antioxidants. The pathogenesis of CLP is governed by T cells and proinflammatory cytokines that lead to the generation of ROSs, which exert harmful effects on cell components. In this context, antioxidants can prevent pathological changes at the cellular level. Therefore, antioxidant therapies may represent an option in the management of CLP patients. In conclusion, the results of the analyzed studies suggest that oxidative stress is involved in the occurrence of CLP, however further studies to include larger numbers of patients are needed. To the best of our knowledge, this is the first review that brings together studies on oxidative stress in CLP offering new insights into its pathogenesis.

## Figures and Tables

**Table 1:** Summary of the studies included in the review.

Biomarker	Number of CLP Patients	Material	CLP Patients vs. Controls	Pathophysiological Significance	Reference
**NO**, **MDA**, **SOD**, **CAT**	40 patients	Serum	MDA, NO, SOD—higher levels CAT—lower levels	Increased lipid peroxidation and decreased antioxidant defense may be involved in the pathogenesis of CLP	Sezer et al. [41] 2007
**NO**, **MDA**, **SOD**, **CAT**	45 patients	Serum, erythrocytes	NO, SOD, MDA—higher levels CAT—lower levels	The alteration of the balance between prooxidants and antioxidants plays an important role in patients with CLP. No differences were observed depending on the clinical type of LP.	Aly et al. [42] 2010
**Sluc**, **Slum**, **Sind-1**, **h**, **H**, **Sind-2**	16 patients	Skin	Sluc, Slum, Sind-1, h, H, Sind-2—higher levels	Free radicals participate in the development of hyperregeneratory processes in CLP, a decrease in the intensity of local oxidative stress being associated with the normalization of the proliferation process.	Sapuntsova et al. [43] 2011
**GPX**, **vitamin C**, **selenium**, **bilirubin**, **uric acid**	30 patients	Serum	GPX, uric acid, bilirubin, selenium—similar levels Vitamin C—lower levels	Vitamin C may be useful in the treatment of CLP patients.	Barikbin et al. [44] 2011
**NO**, **MDA**, **SOD**, **CAT**	30 patients	Serum, skin	NO, MDA, SOD—higher levels (serum, skin) CAT—lower levels (serum, skin)	The imbalance between oxidants and antioxidants may be involved in the pathogenesis of CLP.	Karim et al. [45] 2012
**SOD**, **MDA**, **GPX**, **GSH**, **NO**	60 patients	Serum	MDA, SOD, NO—higher levels GPX, GSH—lower levels	Oxidative stress may be involved in the pathogenesis of CLP.	Hassan et al. [46] 2013
**Uric acid**	61 patients	Serum	Uric acid—lower levels	Uric acid may be a biomarker in patients with CLP, useful for monitoring the effectiveness of therapy and the evolution of the disease.	Chakraborti et al. [47] 2014
**Ascorbic acid**	77 patients (49 CLP patients, 28 OLP patients)	Urine	Ascorbic acid—lower levels	There is a negative relationship between the ascorbic acid levels and disease duration in patients with LP.	Nicolae et al. [48] 2017
**TLR-4**, **RAGE**, **and HMGB1**	24 patients	Skin	mRNA expression of HMGB1 and TLR-4—similar mRNA expression of RAGE—lower HMGB1 and TLR-4—higher levels in the dermis and lower levels in the epidermis. RAGE—higher levels in the dermis	HMGB1 and TLR-4 may contribute to the inflammatory process observed in CLP. The negative regulation of RAGE in CLP may be involved in its pathogenesis.	de Carvalho et al. [49] 2018
**Bilirubin**, **uric acid**, **albumin**, **iron**, **transferrin**, **ferritin**, **copper**, **ceruloplasmin**, **TAC**	77 patients	Serum	TAC—lower levels bilirubin, uric acid, albumin, iron, transferrin, ferritin, copper, ceruloplasmin—similar levels	Evaluation of serum antioxidants levels may be useful in the management and follow-up of CLP patients.	Georgescu et al. [50] 2018
**MDA**, **triglycerides**	50 patients	Serum	MDA, triglycerides—higher levels	A link between chronic inflammation and oxidative stress may be present in LP patients.	Manasa et al. [51] 2019
**NT**, **TT**, **DS**	81 patients	Serum	NT, TT—higher levels DS—similar levels	Increased levels of serum thiols as a response to oxidative stress may contribute to cell proliferation and progression of LP lesions.	Kalkan et al. [52] 2019
**4-HNE TBARS**, **MDA**, **TAS**, **NT**, **TT**, **DS**	31 patients	Serum	4-HNE, MDA, TBARS—higher levels TAS, NT, TT—lower levels DS—higher levels	4-HNE, TBARS, and MDA might be involved in the development of LP lesions by exceeding the tissue antioxidant defense.	Mitran et al. [53] 2019

MDA-malondialdehyde, 4-HNE-4-hydroxynonenal, TBARS-thiobarbituric acid reactive substances, 8-OHdG-8-hydroxy-2′-deoxyguanosine, RAGE-receptor for advanced glycation end-products, NT-native thiol, TT-total thiol, DS-disulphide, NO-nitric oxide, GSH-glutathione, TLR-toll-like receptor, HMGB-1-high mobility group box 1 protein, Sluc-superoxide anion radicals, Slum-hydroxyl radicals, Sind-1-peroxide radicals, h-the concentration of lipid peroxides, H-peroxidation resistance of the substrate, Sind-2-activity of antioxidant antiradical defense, SOD-superoxide dismutase, CAT-catalase, GPX-glutathione peroxidase, TAS-total antioxidant status.
